# Response surface methodology (RSM) modeling to improve removal of ciprofloxacin from aqueous solutions in photocatalytic process using copper oxide nanoparticles (CuO/UV)

**DOI:** 10.1186/s13568-018-0579-2

**Published:** 2018-03-28

**Authors:** Nahid Khoshnamvand, Ferdos Kord Mostafapour, Amir Mohammadi, Maryam Faraji

**Affiliations:** 1 Nutritional Health Research Center, Lorestan University of Medical Sciences, Khorramabad, Iran; 2 Health Promotion Research Center, Zahedan University of Medical Sciences, Zahedan, Iran; 3 Student Research Committee, Faculty of Health, Shahid Sadoughi University of Medical Sciences, Yazd, Iran; 4 Young Researchers and Elite Club, Tehran Medical Sciences Branch, Islamic Azad University, Tehran, Iran

**Keywords:** Antibiotics, Copper oxide nanoparticles, Emerging pollutants, Central composite design, Advanced oxidation processes (AOPs)

## Abstract

Ciprofloxacin (CIP) antibiotic is considered as an emerging and biological resistant pollutant. This study aimed to improve of the removal of CIP from synthetic aqueous solutions in photocatalytic process through copper oxide nanoparticles as catalyst (CuO/UV). The effect of CIP concentration (10–200 mg/l), catalyst dosage included CuO (0.01–0.1 g/l) and pH (3–11) as independent variables on the COD removal efficiency as response in photocatalytic process using UV-C lamps with three different powers of 8, 15 and 30-W were optimized through the central composite design in response surface method using design-expert software. A second order model was selected as the best model with R^2^ values and lack of fit as 0.85 and 0.06 for lamp 8-W, 0.89 and 0.11 for lamp 15-W, and 0.86 and 0.19 for lamp 30-W, respectively. Optimum conditions were obtained in CIP concentration of 11.2 (mg/l), CuO dosage of 0.08 (g/l), and pH value of 8.17. In this condition, predicted maximum COD removal was respectively found 83.79, 93.18, and 98.90% for lamps 8, 15 and 30-W. According to the results, photocatalytic process using copper oxide nanoparticles can effectively compose CIP in aqueous solutions.

## Introduction

Antibiotics, especially fluoroquinolones, have been considered as the important emerging pollutants in water sources and municipal wastewater (Guo et al. 2013). They are priority pollutants due to the high toxicity for algae and bacteria in trace concentration (Hernando et al. 2006). These compounds are extensively used to prevent or treat bacterial infections in humans, animals and plants (Balarak et al. 2017). Antibiotics use in modern aquacultures in diverse areas including Iran in large scale to prevent or treat the infectious diseases in fishes (Adel et al. 2017). The WHO has declared that widespread application of antibiotic in the aquacultures may cause risks for the consumer contributing to the antibacterial resistance in human and veterinary medicine due to the accumulation of their residues in edible tissues of fish (Adel et al. 2017; Conti et al. 2015). Also, antibiotics can release into the surrounding waters during treatment of fish stocks and cause some environmental problems (Adel et al. 2017). Furthermore, presence of antibiotics in the aquatic environments may pose toxicological effects on non-target organisms, disturb the biological balance and photosynthetic cycles of plants (Rakshit et al. 2013).

Ciprofloxacin (CIP) as the most common fluoroquinolones is broadly consumed (Dodd et al. 2006). CIP with k_bio_ = 0.02–0.55 l/gMLSS day appeared not to be easily biodegraded during biological wastewater treatment processes (Tran et al. 2017). Mean concentration of CIP is reported 2.5 μg/l in the hospital wastewater effluent, 0.6 μg/l in influents of the municipal wastewater treatment plant and 14–42 ng/l in the surface waters (Watkinson et al. 2009; El-Shafey et al. 2012). Therefore, wastewater treatment plants should be upgraded with novel unites included effective physiochemical processes such as coagulation, advanced oxidation processes (AOPs) and adsorption with activated carbon or nanoparticles to removal of CIP (Rakshit et al. 2013; Sui et al. 2012; Khoshnamvand et al. 2017).

In previous studies, the degradation and removal of CIP have been reported using hazelnut shell activated carbon (Balarak et al. 2016), magnetite (Fe_3_O_4_(s)) (Rakshit et al. 2013), ozonation and sonolysis (Vasconcelos et al. 2009; Paul et al. 2010), UV/TiO_2_ (Paul et al. 2010), visible light/TiO_2_ (Paul et al. 2007), UV and UV/H_2_O_2_ (Guo et al. 2013). Researches demonstrated that physical processes not be able to effectively remove CIP. On the other hand, chemical processes may generate harmful by-products (Shi et al. 2013). So, AOPs have been introduced as the effective methods to degradation and elimination of antibiotics and other organic compounds. Recent studies were reported a high removal efficiency for CIP through photocatalytic process with semi-conductors such as Tio_2_–ZnO (Skoumal et al. 2006; Norzaee et al. 2017). The mechanism of AOPs is radiation of UV to a semi-conductor material and, consequently, the electron excitation and its emission from the valence band to conduction band. This excitation results in production of active hydroxyl radical (OH·) that effectively oxidize organic pollutants (Xiao et al. 2015). Among the semi-conductors, copper oxide (CuO) nanoparticles have been considered as the high-efficiency catalysts from 1990 due to the extremely effective surface area and more effect of quantum size compared to masses of copper (Han et al. 2006).

Response surface methodology (RSM) is a collection of mathematical and statistical methods to set the experimental models, in which, two stages are essential, the estimation of function and the experimental design. RSM was effectively applied in the experimental studies. The application of RSM is to control the cost of analytical methods and related numerical noise (Dehghani et al. 2017; Amiri et al. 2018; Khayet et al. 2011).

This study aimed to improve of the removal of CIP from synthetic aqueous solutions in photocatalytic process through copper oxide nanoparticles as catalyst (CuO/UV) using UV-C lamps with power of 8, 15 and 30-W. To the best of our knowledge, present study is first study considering CuO nanoparticles as catalyst to removal of CIP from solutions in photocatalytic process.

## Materials and methods

### Chemical

**Fig. 1:**
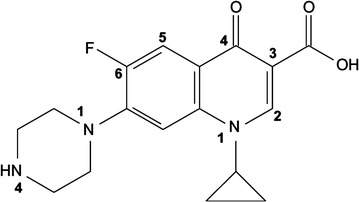
Chemical structure of ciprofloxacin (C_17_H_18_FN_3_O_3_)

**Fig. 2:**
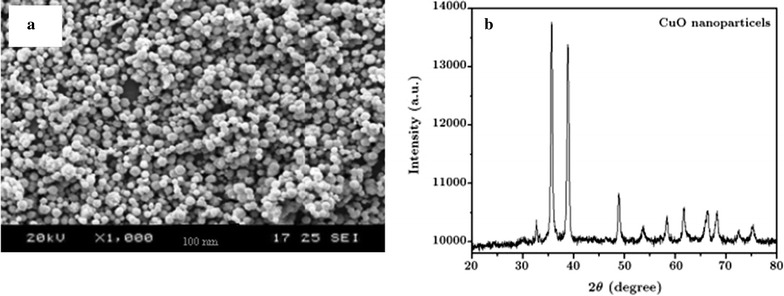
SEM image (**a**) and XRD pattern (**b**) of CuO nanoparticles

In this study, all materials included Ciprofloxacin hydrochloride, C_17_H_18_FN_3_O_3_, (≥ 98%) (Fig. 1), sodium hydroxide (NaOH), hydrochloric acid (HCl), copper oxide (CuO) nanoparticles (size > 50 nm, molecular weight: 79.55 g/mol, purity ≥ 99%) were purchased from Sigma-Aldrich Company. Also, UV-C lamps with power 8, 15 and 30-W and wavelength of 254 nm were bought from Philips company (Germany). The surface textural and morphological structure of the CuO nanoparticles were analyzed with a scanning electron microscope (SEM) (HITACHI Model S-4160). Also, CuO nanoparticles was analyzed by X-ray diffraction (XRD) (Philips, Model XPERT PW 3040/60). SEM image and XRD pattern are shown in Fig. 2a, b, respectively.

### Experimental design

The effect of independent variables mentioned in Table 1 on the dependent variable or response (COD removal efficiency) and the optimum conditions were investigated using the central composite design (CCD) in RSM. A second-order model can be efficiently constructed with CCD. CCD is actually a first-order (2 N) designs that amplified by center and axial points to estimation of the second-order model parameters (Amiri et al. 2018). p-value less than 0.05 was considered significant in all statistical analyses. At first, a factorial design was done to determine significant variables. Then, experiments were designed based on Montgomery method (Myers et al. 2016) in a central composite rotatable design for mentioned independent variables in 20 runs.

**Table 1:** Independent variables and their ranges

Independent variables	Symbol	Levels of variables
Cipro concentration (mg/l)	X_1_	10–200
CuO dosage (g/l)	X_2_	0.01–0.1
pH	X_3_	3–11

The experimental method results were used to specify an empirical second order polynomial regression model that is shown as follows (Eq. 1):

1 $$Y = \beta_{0} + \mathop \sum \limits_{j = 1}^{K} \beta_{j} x_{j} + \mathop \sum \limits_{j = 1}^{K} \beta_{jj} x_{j}^{2} + \mathop \sum \nolimits \mathop \sum \limits_{i < j = 2}^{K} \beta_{ij} x_{i} x_{j}$$

where Y is the response; x_i_ and x_j_ are independent variables (i and j ranged from 1 to k); β_0_ is the constant term; β_j_ is the linear coefficient, β_ij_ is the interaction coefficient, and β_jj_ is the quadratic coefficient; k is the number of independent variables (k = 3 in this study) (Amiri et al. 2018).

### Batch studies

**Fig. 3:**
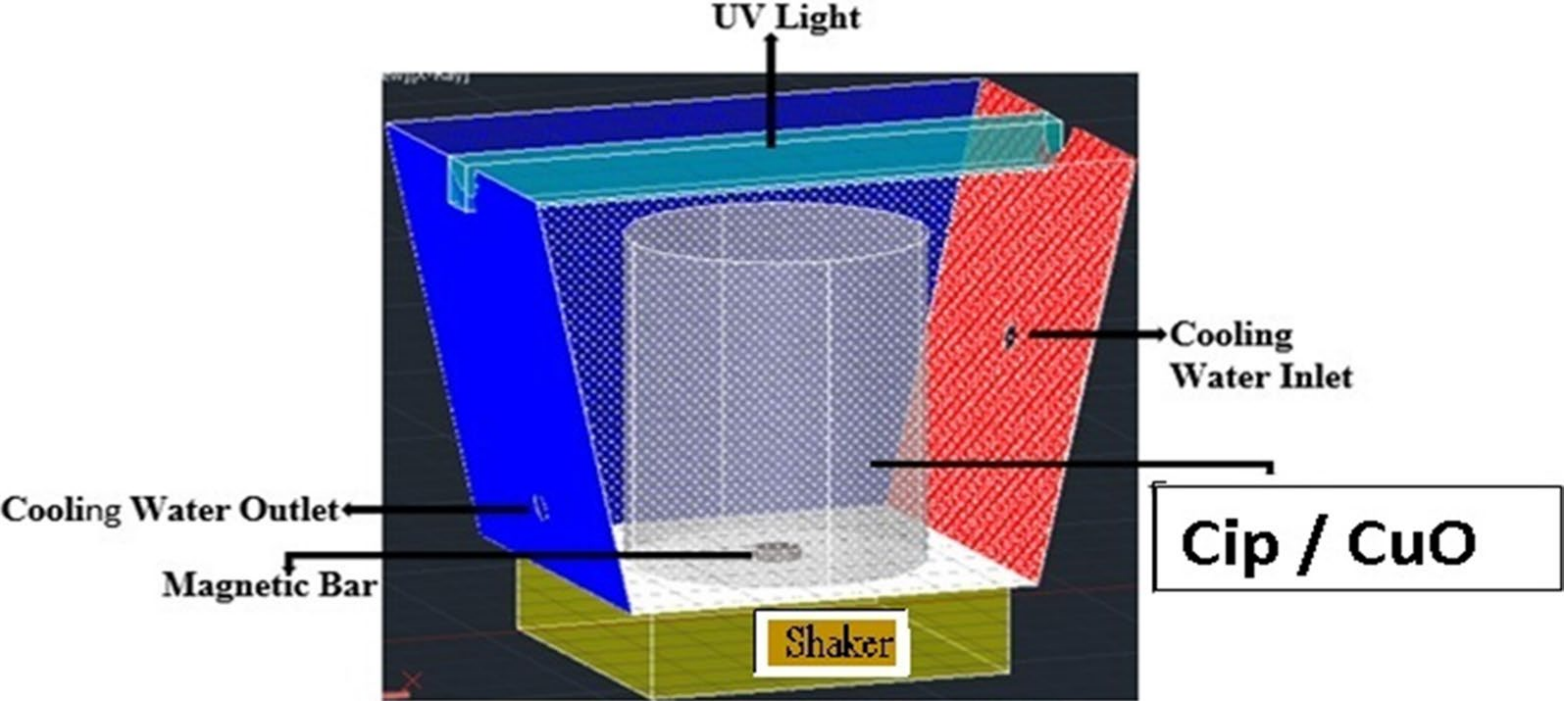
Schematic diagram of photocatalytic reactor

The photocatalytic process was run in a batch Plexiglas reactor (Fig. 3) included two chambers. The main chamber (reaction chamber) had a useful volume of 500 ml. The secondary chamber with a volume of 3 l and a continuous flow of water surrounded the main chamber to keep temperature constant at 27 ± 3 °C. Reactor was equipped with the UV lamp (LU 100A) with different powers, 8, 15 and 30-W. The reactor was wrapped in aluminum foil to prevent UV reflection and increase lamp efficiency. Magnetic stirring was used to homogenization of solution in the reaction reactor. Batch experiments were performed according to the designed runs in a constant time 60 min, triplicate. CIP stock solution was daily prepared and stored at 4 °C. Then, different concentrations of CIP were made from stock in deionized water. The CIP solution and CuO nanoparticles were injected into the reactor. pH of solutions was adjusted by HCl and NaOH, 1 N. Suspension was maintained in the dark for 30 min so the CIP solution and nanoparticles attain equilibrium condition. Nanoparticles in outlet solutions were separated through centrifugation for 10 min at 3000 rpm followed filtration with the 0.22 μm polytetrafluoroethylene syringe filters (Schleicher & Schuell, Germany). COD concentration in inlet and outlet solutions was analyzed using UV/VIS spectrophotometer (HACH, DR-5000) according to the D5220 method in the handbook of standard methods for the examination of water and wastewater (Federation and Association 2005). COD removal efficiency (Y) was calculated from Eq. 2:

2 $$Y\left( \% \right) = C_{0} - C_{e} /C_{0} \times 100$$

where C_*e*_ and C_0_ are COD concentrations in inlet and outlet solutions (mg/l), respectively.

### Statistical analysis

Multiple regression analysis through the generalized least square was used to analyze the experimental data and explore the relationship between the independent variables and response using the design-expert software (version-10). Two-way analysis of variance (ANOVA) test was used to compare the mean difference of COD removal between independent variables. Results were defined statistically significant if p-value was lower than 0.05.

## Results

### Model fitting and statistical analysis

Independent variables and COD removal percent in form of experimental and predicted responses in 20 runs for the development of mathematical equations were represented in Table 2. Different regression models were analyzed and finally a second order model was fitted, as the best model for COD removal percent, between the experimental results obtained on the basis of the central composite experimental design and the input variables. COD removal efficiency was assessed as a function of CIP concentration (X_1_), CuO nanoparticles dosage (X_2_) and pH (X_3_) and calculated as the sum of a constant, three first-order effects (X_1_, X_2_ and X_3_), three interaction effects (X_1_X_2_, X_1_X_3_ and X_2_X_3_) and three second-order effects (X_1_^2^, X_2_^2^ and X_3_^2^). The model fitness was verified by the correlation coefficient R^2^ of the model and p-value for lack of fit, the R^2^ values were gained 0.85, 0.89 and 0.86 for lamps 8, 15 and 30-W, respectively. p-value for lack of fit values was found higher than 0.05 for all three models. The F values as 5.98, 3.72 and 2.61 and p-values as 0.03, 0.01 and 0.009 were calculated respectively for 8, 15 and 30-W lamps (Tables 3, 4, 5).

**Table 2:** Central composite design (CCD) and observed responses

Run order	Actual variables			Response (%)					
				Experimental			Predicted		
	Cipro	CuO	pH	Lamp 8 W	Lamp 15 W	Lamp 30 W	Lamp 8 W	Lamp 15 W	Lamp 30 W
1	105.0	0.06	3.0	3.3	7.1	11.9	3.1	5.9	12.5
2	48.5	0.03	4.6	8.9	9.9	17.8	14.6	14.3	20.5
3	48.5	0.08	4.6	47.9	53.8	68	51.8	58.5	70.9
4	161.5	0.03	4.6	19.1	23.2	23.1	12.4	17.8	17.6
5	161.5	0.08	4.6	48.2	58.3	69.2	42.6	54.4	64.5
6	10.0	0.06	7.0	82.3	85.1	87.2	68.6	73.2	78.2
7	105.0	0.01	7.0	25.5	32.4	37	25.1	32.9	38.1
8	105.0	0.06	7.0	46.8	50.8	62.8	51.2	53.0	64.1
9	105.0	0.06	7.0	45.7	60.2	69.3	51.2	53.0	64.1
10	105.0	0.06	7.0	45.4	48.9	58.9	51.2	53.0	64.1
11	105.0	0.06	7.0	58.3	45.9	55.8	51.2	53.0	64.1
12	105.0	0.06	7.0	48.7	52.8	67.2	51.2	53.0	64.1
13	105.0	0.06	7.0	56.8	47.3	57.3	41.9	37.0	46.9
14	105.0	0.10	7.0	59.5	68.2	83.5	59.9	66.8	83.8
15	200.0	0.06	7.0	21.8	27.9	32.9	35.4	38.6	42.7
16	48.5	0.03	9.4	38.7	44.1	48.8	42.7	47.8	51.2
17	48.5	0.08	9.4	59.7	65.4	75.9	64.9	70.4	79.1
18	161.5	0.03	9.4	29.2	35	40	23.5	29.7	35.8
19	161.5	0.08	9.4	46.1	50.4	65.3	38.7	44.8	60.2
20	105.0	0.06	11.0	29.4	36	46	29.9	36.8	46.4

**Table 3:** Analysis of variance (ANOVA) results for the fitted polynomial model for COD removal using photocatalytic process with lamp 8-W

Lamp 8	Sum of squares	df	Mean square	F value	p-value
Cipro concentration	607.65	1	607.65	5.32	0.05
CuO dosage	2066.08	1	2066.08	18.09	0.00
pH	623.99	1	623.99	5.46	0.04
pH^2^	1453.75	1	1453.75	12.73	0.01
Residual	1027.80	9	114.20		
Lack of fit	906.60	5	181.32	5.98	0.06
Pure error	121.20	4	30.30		

Adjusted R^2^: 0.8530, p-value: 0.0280

**Table 4:** Analysis of variance (ANOVA) results for the fitted polynomial model for COD removal using photocatalytic process with lamp 15-W

Lamp 15	Sum of squares	df	Mean square	F value	p-value
Cipro concentration	370.40	1	370.40	4.61	0.04
CuO dosage	2646.92	1	2646.92	32.96	0.00
pH	1054.37	1	1054.37	13.13	0.01
pH^2^	899.84	1	899.84	11.21	0.01
Residual	722.71	9	80.30		
Lack of fit	594.71	5	118.94	3.72	0.11
Pure error	128.00	4	32.00		

Adjusted R^2^: 0.8961, p-value: 0.0178

**Table 5:** Analysis of variance (ANOVA) results for the fitted polynomial model for COD removal using photocatalytic process with lamp 30-W

Lamp 30	Sum of squares	df	Mean square	F value	p-value
Cipro concentration	359.08	1	359.08	5.46	0.04
CuO dosage	4212.77	1	4212.77	64.02	< 0.0001
pH	1254.09	1	1254.09	19.06	0.0018
pH^2^	1095.90	1	1095.90	16.65	0.0028
Residual	592.28	9	65.81		
Lack of fit	453.48	5	90.70	2.61	0.19
Pure error	138.80	4	34.70		

Adjusted R^2^: 0.8618, p-value: 0.0096

The coefficients of the models for the response were estimated using multiple regression analysis technique included in the RSM. The second order models obtained in terms of actual factors for significant coefficients (p-values < 0.05) were given as follows by Eqs. 3–5. The insignificant coefficients (p-values > 0.05) were removed from the model.

3 $$\begin{aligned} Y\,(COD\;Removal\;(\% ))\;lamp\;8\;{\text{W}} = & - \,142.83\, + \,0.03\,*\,CIP\;concentration\, \\ &+ \,1243.72\,*\,{\text{CuO}}\;dosage \\ & + \,37.37*pH - \,1.88\,*\,pH^2\\ \end{aligned}$$

4 $$\begin{aligned} Y\,(COD\;Removal\,(\% ))\;lamp\;15\;{\text{W}} = & - \,137.17\, + \,0.1\,*\,CIP\;concentration\, \\ &+ \,1011.69\,*\,{\text{CuO}}\;dosage \\ & + \,35.40\,*\,pH - \,1.48\,*\,pH^2\\ \end{aligned}$$

5 $$\begin{aligned} Y\,\left( {COD\;Removal\,\left( \% \right)} \right)\;lamp\;30\;{\text{W}} = & - \,123.83\, - \,0.02\,*\,CIP\;concentration\, \\ &+ \,1084.68\,*\,{\text{CuO}}\;dosage \\ & + 36.53\,*\,pH - \,1.63\,*\,pH^2\\ \end{aligned}$$

where X_1_, X_2_ and X_3_ are CIP concentration, CuO nanoparticles dosage and pH, respectively.

### Effect of various parameters on COD removal efficiency

Results of ANOVA test for three lamps, 8, 15 and 30-W, are presented in Tables 3, 4 and 5. The COD removal was graphically shown through contour plots. Graphs were plotted as the effect of two variables on the removal efficiency that vary within the determined experimental ranges, keeping one of variables at a fixed level (central level).

### Effect of CIP concentration

**Fig. 4:**
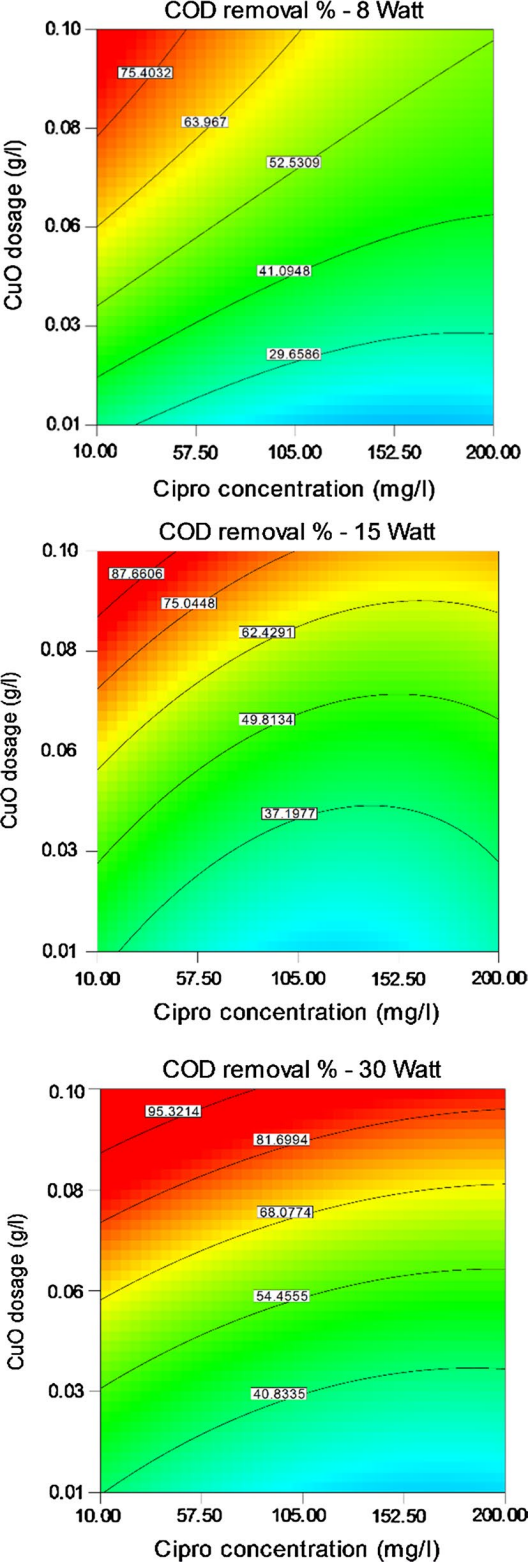
Effect of Cipro concentration and CuO dosage on COD removal efficiency

Figure 4 compares the effect of CIP concentration and CuO dosage on the COD removal efficiency at central level of pH equal to 7 for three lamps. Optimum CIP concentration was obtained 11.2 mg/l the based on optimization data results (Table 6).

**Table 6:** Optimization results for independent variables and responses in predicted and experimental values

Optimum condition			COD removal (%)					
			Predicted			Experimental^a^		
Cipro conc.	CuO dosage	pH	Lamp 8 W	Lamp 15 W	Lamp 30 W	Lamp 8 W	Lamp 15 W	Lamp 30 W
11.2	0.08	8.17	83.79	93.18	98.90	82.23 ± 1.23	92.96 ± 1.47	96.58 ± 1.04

^a^Mean ± standard deviation (n = 3)

### Effect of CuO dosage

COD removal efficiency according to the CuO dosage in different concentrations of CIP in Fig. 4 demonstrated that addition of CuO improved COD removal efficiency, but according to optimum CuO dosage as 0.08 g/l (Table 6), COD removal was decreased in the CuO dosage higher than 0.08 g/l.

### Effect of solution pH

**Fig. 5:**
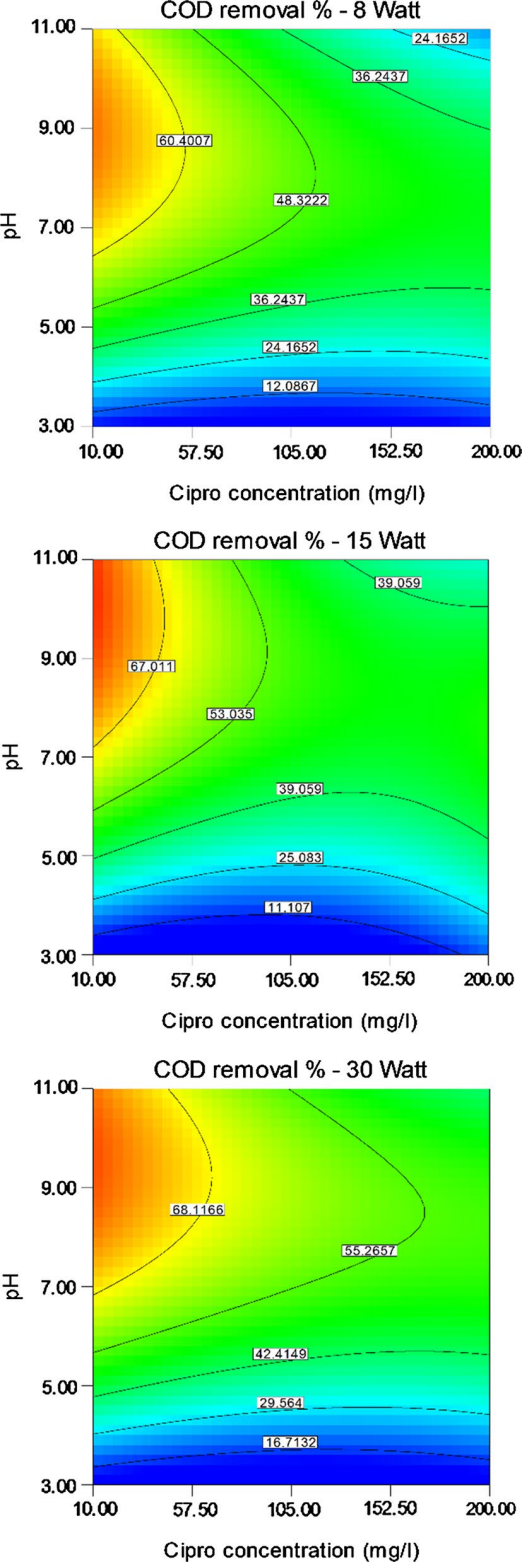
Effect of Cipro concentration and solution pH on COD removal efficiency

**Fig. 6:**
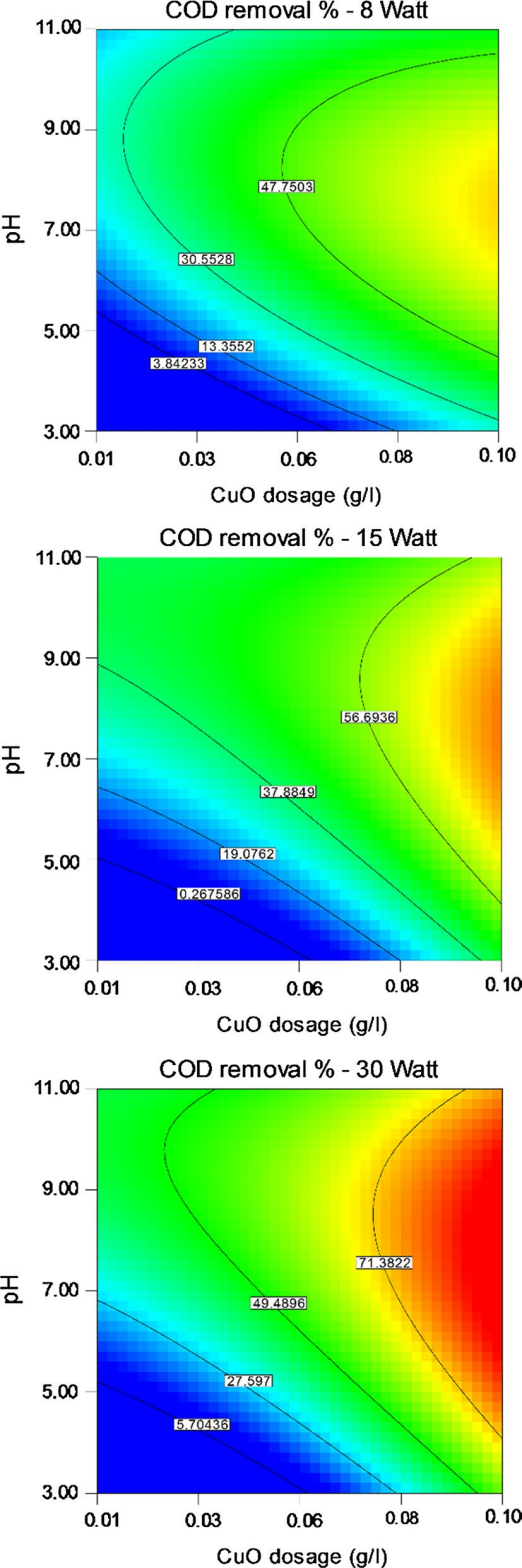
Effect of CuO dosage and solution pH on COD removal efficiency

The effect of solution pH on the COD removal in different concentrations of CIP and CuO is presented in Figs. 5 and 6, respectively. It is clearly shown that the performance of CuO/UV process is dependent of pH and increased efficiency was observed in the pH range of 3–8. Also, efficiency was decreased in pH values higher than 8. The pH value equal to 8 was found as optimum pH in this study (Table 6).

### Validation of the model

Based on the optimization results using the numerical method in Table 6, the maximum efficiencies for COD removal were obtained as 83.79, 93.18 and 98.90% by the lamps of 8, 15 and 30-W, respectively at CIP concentration 11.2 (mg/l), CuO dosage 0.08 (g/l) and pH value 8.17. The experimental removal efficiencies for COD were 82.23 ± 1.23, 92.96 ± 1.47 and 96.58 ± 1.04% in three experiments based on optimum condition, corresponding well to the predicted efficiencies, that approved that the models were adequate for the optimization of variables.

## Discussion

Second order models were fitted between the experimental results of COD removal obtained on the basis of the central composite experimental design and the independent variables in Table 2. The R^2^ values of models obtained as 0.85, 0.89 and 0.86 for lamps 8, 15 and 30-W, respectively were shown that there was a high correlation between predicted values from the fitted model and experimental data points.

However, a high value of R^2^ does not mean that the model is the best. For this reason, the variances were applied to measure the lack of fit between the predicted and the experimental data. Lack of fit value is calculated using the difference between of sum of the squares for the experimental response variable and the its predicted values by the model (Yaghmaeian et al. 2016; Amiri et al. 2018). Based on the p-value for lack of fit values, there is no significant difference between experimental and predicted model data, so it can be concluded the models has a good prediction for COD removal. F values greater than unity and p-values higher than 0.05 for three lamps were approved that the fitted models were significant (Tables 3, 4, 5). According to Eqs. 3–5, CIP concentration, CuO nanoparticles dosage and pH were important factors in the COD removal process related to the photocatalytic degradation of CIP drug in aqueous solutions using CuO nanoparticles. According to the model coefficients, the linear term of CuO nanoparticles dosage and after that, pH had the largest effect on COD removal in photocatalytic process.

It was determined by ANOVA test that mean of COD removal percent was significantly different between variables of CIP concentration, CuO nanoparticles dosage and pH in the defined range for all three lamps.

According to Fig. 4, COD removal efficiency was decreased by increased CIP concentration from optimum value equal to 11.2 mg/l. This finding could be attributed to the more time needed to decompose of CIP when a more concentration of CIP be exposed to UV. Also, CIP acts as a barrier and suppresses UV penetration to the suspension (Guo et al. 2013). On the other hand, increasing CIP concentration can be led to adsorption of irradiated UV by CIP molecules and reduction of COD removal efficiency (Kümmerer 2003). This finding was accordance to the other studies (El-Sayed et al. 2014; Guo et al. 2013, Mostafapour et al. 2016).

The amount of catalyst greatly affected the degradation rate of compounds as if addition of CuO improved COD removal efficiency (Fig. 4), but efficiency was decreased in the values of CuO dosage greater than optimum point as 0.08 g/l. An overdose of the catalyst decreases process efficiency through decreasing the UV penetration due to turbidity associated to the excess catalyst clusters, diffusing UV radiation and reducing the total surface area that can be stimulated (Alimoradzadeh et al. 2012). This result was similar to other researches (Mostafapour et al. 2016; Alimoradzadeh et al. 2012).

The pH is an important parameter in removal of pollutants from aqueous solutions (Faraji et al. 2017). Maximum efficiency was gain in pH value of 8 and after that increased pH resulted in decreasing efficiency. The pH affects AOPs through effects on the rate of chemical reactions and production of radicals in the process (Pouran et al. 2014). Also, pH can change the surface charge properties of the photocatalyst and possibly the chemical structure of the CIP; so solution pH affects photocatalyst reactions. At low pH values, high concentration of protons delayed the photodegradation of CIP under UV light. Protons had high affinity for the hydroxyl anion, preventing the production of hydroxyl radicals. Anionic form of CIP can be generated in solutions with pH values greater than 7.7 (El-Kemary et al. 2010). Bobu et al. concluded that very high pH values lead to increasing HO_2_^−^ and consumption of OH radicals by carbonate and bicarbonate ions (Bobu et al. 2008). At pH less than 5.7 and higher than 9.4, the tendency of the two substances CIP and CuO nanoparticles may reduce due to the neutrality of the surface charge of two substances to each other, and recovery can reduce. Between these two pH values, the removal efficiency would be increased (El-Kemary et al. 2010).

Our study suggests that advanced oxidation process using copper oxide nanoparticles as the high-efficiency catalysts and UV lamps be able to mineralization of antibiotics such as ciprofloxacin that are biological resistant pollutants.

## Abbreviations

CIP: ciprofloxacin
AOPs: advanced oxidation processes
CuO: copper oxide
RSM: response surface methodology
XRD: X-ray diffraction
COD: chemical oxygen demand
CCD: central composite design
ANOVA: analysis of variance
